# Preventive Treatments of Iron Deficiency Anaemia in Pregnancy: A Review of Their Effectiveness and Implications for Health System Strengthening

**DOI:** 10.1155/2012/454601

**Published:** 2012-07-10

**Authors:** Kayode O. Osungbade, Adeolu O. Oladunjoye

**Affiliations:** ^1^Department of Health Policy and Management, Faculty of Public Health, College of Medicine and University College Hospital, University of Ibadan, P.M.B. 5017 General Post Office, Ibadan, Nigeria; ^2^Department of Community Medicine, University College Hospital, P.M.B. 5116 General Post Office, Ibadan, Nigeria

## Abstract

*Objectives*. We conducted a review of effectiveness of preventive treatments of iron deficiency anaemia in pregnancy in developing countries and highlighted their constraints as well as interventions required to strengthen the health services. *Methods*. Literature from Pubmed (MEDLINE), AJOL, Google Scholar, and Cochrane database was reviewed. *Results*. Evidence-based preventive treatment options for iron deficiency anaemia in pregnancy include prophylaxis iron supplements and food fortification with iron. Evidence abounds on their effectiveness in reducing the prevalence of iron deficiency anaemia in pregnancy. However, these prospects are threatened by side effects of iron supplements, low utilization of maternal health service in developing countries, partial implementation of preventive treatments, and weak infrastructure and political commitment to implement mass fortification of local staple foods by national governments. *Conclusion*. Sustainability of effectiveness of preventive treatments of iron deficiency anaemia in pregnancy could be achieved if the identified threats are adequately addressed.

## 1. Introduction

Iron deficiency anaemia is defined as anaemia accompanied by depleted iron stores and signs of a compromised supply of iron to the tissues [1]. There is variation in haemoglobin levels during pregnancy; at the beginning of a pregnancy, there is a normal reduction in haemoglobin level followed by a slight rise towards the end of pregnancy [2]. The initial reduction has been explained to result from increased red cell mass and demands of the fetus which exceeds iron intake with consequent reduction in iron stores of the woman's body [2]. Thus, the World Health Organization has defined anaemia in pregnancy as a haemoglobin value below 11 g/dL [1, 3].

There are two known factors which contribute to development of iron deficiency anaemia (IDA) in pregnancy; the first is the woman's iron stores at the time of conception and the second is the amount of iron absorbed during gestation. The fact that anaemia frequently does occur in pregnancy among women in developing countries is an indication that preexisting iron stores are often inadequate and physiological adaptations to pregnancy are insufficient to meet the increased requirements [4]. Hence, iron supplementation in pregnancy has become a standard and routine practice as a preventive treatment for iron deficiency anaemia in pregnancy in developing countries. In view of the foregoing, a review of effectiveness of preventive treatments of iron deficiency anaemia in pregnancy was conducted; furthermore, constraints were highlighted and suggestions for improvement were provided. 

## 2. Methods

We conducted a review of literature on evidence-based preventive treatments of iron deficiency anaemia in pregnancy with particular reference to developing countries. The following search terms were used: prevalence, burden, iron deficiency, anaemia in pregnancy, preventive treatments, and developing countries. Cross-sectional, observational, and randomized control trials' literature on the subject published between 2000 and 2011 served as the main sources of information. These works of literature were obtained from the commonly used medical databases such as PubMed (Medline), AJOL, and Google Scholar; in addition, Cochrane Library was used as source for systematic reviews on the subject matter.

The search generated 27 related articles in the following categories: prevalence—2, treatments—8, reviews—6, interventions/trials—6, dosage—1, and technical reports—4. This paper was limited to the six randomised and quasi-randomised trials (listed in Table 1) on preventive treatments of iron deficiency anaemia in pregnancy, which met at least one of the following inclusion criteria: (i) comparison between daily routine oral supplementation with iron or ironfolic acid and no supplementation/placebo; (ii) comparison between daily routine oral supplementation with iron or ironfolic acid and routine-intermittent (weekly and twice weekly) regimens; (iii) comparison between intermittent oral iron or ironfolic acid supplementation and no supplementation/placebo; (iv) comparison between intravenous route versus oral route of iron supplementation; (v) comparison between food fortification with iron and no fortification/placebo.

## 3. Results

### 3.1. Prevalence and Burden of Iron Deficiency Anaemia in Pregnancy in Developing Countries

Worldwide, anemia affects over two billion people and the World Health Organization (WHO) has estimated that half of these are due to iron deficiency [5, 6]. Iron deficiency is not only the most prevalent but also the most neglected nutrient deficiency in the world, particularly among pregnant women and children in developing countries [7]. Presently, over 40 million pregnant women suffer from iron deficiency (ID) and its consequences in developing countries [8]. 

Iron deficiency is the most common cause of anaemia in pregnancy [2]. Iron deficiency anaemia accounts for 75–95% of cases of anaemia in pregnancy [9]. Iron-deficiency anaemia, the late manifestation of chronic iron deficiency, is thought to be the most common nutrient deficiency among pregnant women [10]. Studies conducted on pregnant women in Zimbabwe, China, India, and Mexico from 1996 to 2008 indicated that between 43% and 73% of the women were iron deficient (usually diagnosed as a low-ferritin concentration); out of these, 7% to 33% had IDA [4]. 

Among pregnant women, IDA has been associated with increased risks of low birth weight, prematurity, and maternal morbidity [11]. UNICEF has reported deaths of an estimated 50,000 young women per year globally in pregnancy and childbirth due to severe iron deficiency anemia [12]. The high frequency of iron deficiency anaemia in the developing countries has substantial health and economic cost implications. An analysis of 10 developing countries reported $0.32 per head or 0.57% of gross domestic product as a median value of physical productivity loss per year resulting from iron deficiency [13]. 

### 3.2. Evidence-Based Preventive Treatment Options

#### 3.2.1. Prophylaxis Iron Supplements

The high physiological requirement for iron in pregnancy is difficult to meet with most diets; this is so especially in developing countries where food requirement is a problem. During pregnancy, iron requirements are not uniform [14]. In the first trimester, daily needs decrease due to the absence of menstruation; thereby saving about 0.56 mg of iron per day, or 160 mg for the pregnancy [15]. In the second trimester, blood volume increases by 45% with an increase in plasma volume of 50%; red cell mass is raised by 35% which amounts to about 450 mg of iron in a 55 kg woman [4]. Fetal demands for iron are maximal during the third trimester and these are estimated at about 270 mg in a 3 kg fetus [16]. Therefore, an average daily dose of 4–6 mg of iron is required in the second and third trimesters of pregnancy [14]. 

Overall, a woman requires about 2–2.8 mg of iron per day during pregnancy [17]. But she will need to consume much more to obtain this daily requirement (i.e., between 20 and 48 mg of dietary iron) [18]. However, literature has presented different views on iron requirements during pregnancy ranging from 450 to 1,150 mg with a median of 790 mg [14, 19–21]. Since these requirements are difficult to meet through an ordinary diet, especially in developing countries where most diets do not contain adequate bioavailable iron, routine iron supplementation in pregnancy has been found to be of immense benefit [14, 19–21].

Iron deficiency in nonpregnant populations can be measured quite precisely using laboratory tests such as serum ferritin, serum iron, transferrin, transferrin saturation, and transferrin receptors [7]. However, there is a limitation to the use of some of these biochemical indicators of iron status in certain settings and conditions; thus, necessitating caution in their interpretations. For example, serum ferritin level, being an acute-phase protein, is raised in associated underlying infections and long-term diseases; it can therefore give a false normal or high level in a state of iron deficiency. Whereas in pregnancy, serum ferritin levels decline even in women ingesting daily supplements of iron [22–24]. 

There are different forms of preventive treatment of iron deficiency anaemia in pregnancy. Iron supplements can be given by mouth and parenteral route as intramuscular and intravenous injections; in addition, it can be administered as blood transfusion and recombinant erythropoietin with iron. The first choice in the prophylaxis of iron deficiency anaemia for almost all women is oral iron replacement because of its effectiveness, safety, and low cost [25]. Oral iron, either as iron sulphate or fumarate, is the most commonly used preventive treatment for iron deficiency and iron deficiency anaemia in pregnancy. Direct iron supplementation has been extensively used in most developing countries as an intervention to prevent iron deficiency and consequently anaemia during pregnancy [7]. Thus, preventive treatment with iron supplements has always been given in combination with folic acid and this has been included as part of routine antenatal care provided to pregnant women at every visit in developing countries [26]. The rationale for this combination is to meet increased folic acid requirements in pregnancy brought about by the rapidly dividing cells in the fetus and elevated urinary losses [7]. 

The International Nutritional Anaemia Consultative Group had recommended a daily dose of 60 mg of iron for pregnant nonanaemic women, if supplementation for more than six months is possible before delivery [27]. An increased daily dose of 120 mg of iron is further suggested if the duration of supplementation in pregnant nonanaemic women is shorter or where the prevalence of iron deficiency in women is high or in settings where pregnant women are generally anaemic [27]. This supplement should include 400 *μ*g of folic acid or lower doses, if this amount is not available [7]. 

#### 3.2.2. Food Fortification with Iron

Iron fortification involves the addition of iron, usually with folic acid, to an appropriate food vehicle that is made available to the population at large. Food fortification with iron has thus become a promising approach for preventing iron deficiency anaemia in pregnancy in developing countries. Iron fortification of foods might be particularly useful and cost effective in settings where the logistics of oral iron supplementation among pregnant women are highly challenging. In addition, it is found very useful in developing countries where the rate of compliance with preventive treatment of iron is poor [4]. 

To this end, a variety of food items such as cereal flour (maize or wheat), salt, beverage, milk, sugar, noodles, rice, and fish sauce had been fortified with iron and used successfully as dietary supplements to boost iron stores, and hence improve haemoglobin levels in the population [28–31]. Elemental iron powders are the most widely used iron compound in fortification programmes since about 50 years [32]. 

### 3.3. A Review of Effectiveness of Preventive Treatments of Iron Deficiency Anaemia in Pregnancy

#### 3.3.1. Prophylaxis Iron Supplements

The overall impact of interventions on iron supplementation under field conditions has been limited and its effectiveness questioned [33]. These concerns had been attributed to inadequate infrastructure and poor compliance with preventive treatment, among others [34]. Furthermore, the effectiveness of this intervention has been evaluated mostly in terms of improvement in haemoglobin concentration, rather than maternal or infant health [35]. For example, a randomized controlled trial among pregnant women in Switzerland showed that the parenteral route of iron prophylaxis of anaemia has no clinically significant benefit over oral route as there was no significant difference in maternal outcomes and serious adverse events between the two groups [25]. 

A Cochrane review of a randomized control trial conducted in Pakistan reported that daily iron treatment is better than intermittent iron supplementation in increasing haemoglobin level at delivery among pregnant women in developing countries [2]. Findings from other studies on routine daily or weekly antenatal iron or iron plus folic acid supplementation showed that it may be of benefit, especially where pregestational iron deficiency and anaemia are prevalent [7]. A recently published randomized study found no difference in most pregnancy outcomes between daily and twice weekly iron supplementation regimens, though the daily regimen was found to be more effective than twice weekly regimen in preventing Hb decrement at near term [8]. The timing and dosage of oral iron are also controversial as most studies have focused on preventive treatment from midpregnancy, at or before 20 weeks gestation [4]. 

Some researchers, on the other hand, believe that both daily and weekly iron supplementation are relatively unsuccessful in the reduction of prevalence of anemia in pregnancy. They opined that sufficient attention should be paid to adolescent girls and women of reproductive age long before pregnancy and suggested intermittent low-dose iron supplements and in some cases, combined with necessary micronutrients [36, 37]. Apart from its effectiveness, it was argued that intermittent supplementation is more physiological by avoiding mucosal absorption block and excessive pooling of intestinal iron with associated oxidative stress; furthermore, it has logistic advantages of distribution particularly in areas of limited supply and many of the gastrointestinal side effects of daily iron are avoided [4]. 

Experiences on parenteral iron use are predominantly, but not exclusively, from the developed world as reports of its prophylactic use in pregnancy are scant; this may be related to concerns about adverse reactions associated with its use in parenteral form. However, intravenous iron sucrose in particular has been used in several recent studies and might be highly beneficial in refractory patients or those intolerant of oral iron formulations [4]. Though, because of its immediate bioavailability, it may result in a more rapid rise in haemoglobin level in anaemic patients compared with oral iron; but it probably does not confer an advantage in preventing anaemia in pregnancy [25, 38]. 

The WHO technical working group on the prevention and the treatment of severe anaemia has documented that parenteral iron therapy produces a rapid and complete correction of iron deficiency, including replacement of iron stores; thereby producing a more rapid erythropoietic response than oral iron replacement [39]. However, its use should be limited to a selected group of patients who are unable to tolerate oral iron and in whom oral iron therapy fails due to noncompliance. It is also indicated in pregnant women whose hemoglobin level is required to be restored rapidly such as those who present too close to term and those who have severe anemia [40]. 

#### 3.3.2. Food Fortification with Iron

Food fortification with iron has also been shown to be an equally effective strategy of boosting haemoglobin level in the population, including pregnant women. Asibey-Berko et al. in 2007 recorded 19.5% significant increase in the prevalence of anaemia among rural Ghanaian women, who were not exposed to iron-fortified salt [29]. It was also shown that iron fortification of sugar (with mean intake of 4 mg/day) in nonpregnant Guatemalan women over three years resulted in a substantial increase in iron stores, with reserves still increasing by about 40 mg/year after the third year [41]. Furthermore, two Vietnamese studies showed similar improvements in iron stores following ingestion of ironfortified fish sauce for six to 12 months [42, 43]. Comparable results were documented with dietary supplements containing iron and ironfortified milk [30, 31]. 

### 3.4. Constraints to Successful Preventive Treatments of Iron Deficiency Anaemia in Pregnancy

#### 3.4.1. Side Effects of Iron Supplements

Gastrointestinal distress is commonly observed in women consuming high levels of supplemental iron on an empty stomach [7]. Thus, occurrence of gastrointestinal symptoms is usually considered as a critical adverse effect on which a tolerable upper intake level for iron is based for an individual. High dose of oral iron supplements is commonly associated with gastrointestinal effects such as constipation, nausea, vomiting, and diarrhea; the frequency and severity of which vary according to the amount of elemental iron released into the stomach. In addition, elevation of “*free iron*” in the plasma and hence lipid peroxidation which is indicative of oxidative stress has been reported [44–46]. 

Intramuscular or intravenous iron is thought to be associated with allergic reactions and anaphylactic shock; furthermore, parenteral iron is thought to predispose to venous thrombosis and occasionally cardiac arrest and death [2]. Parenteral iron sucrose complex is known to have several advantages because of its low-allergenic properties and consequently, an extremely low incidence of severe side effects such as anaphylactic reactions [47, 48]; however, its use requires caution as it may not be completely devoid of side effect.

Other disadvantages of intravenous iron supplementation include cost and invasiveness of the procedure. However, it is argued that cost benefit of intravenous iron prophylaxis may be large taking into consideration the opportunity costs of erythropoiesis-stimulating agents, blood transfusions, and hospitalization [25]. 

#### 3.4.2. Low Utilization of Maternal Health Service in Developing Countries

It is well established that antenatal care provides pregnant women with opportunities to receive cost-effective interventions which are beneficial to mother and child; these interventions include preventive treatments of iron deficiency anemia. However, the potentials of antenatal service have not been maximally utilized in developing countries. This is because these settings are characterized by poor maternal health service indicators such as nonutilization of service or delayed antenatal visit. 

For example, researchers have reported a common occurrence of unbooked pregnancies [49, 50] and a wide range (60% to 90%) of antenatal care utilization rates (i.e., antenatal care clinic attendance of at least once during most recent pregnancy) [51–53]. In addition, for certain reasons, a substantial proportion (20% to 80%) of pregnant women in these settings make their first antenatal visit in their third trimester [54–56]. 

#### 3.4.3. Partial Implementation of Preventive Treatments

The success of routine iron and folate supplementation, especially in areas with a high prevalence of anemia, recommended as a component of antenatal care package for all pregnant women by the World Health Organization is threatened by the practice of partial implementation of preventive treatments of health workers. Researchers have persistently reported noncompliance with this recommendation at a given antenatal visit. Van Eijk et al. in 2006 reported that 53% and 44% of pregnant women received iron and folate supplementation, respectively, during last pregnancy [54]. Other studies reported 36–54% iron supplementation [51, 55].

#### 3.4.4. Weak Infrastructure and Political Commitment

The efforts of World Food Programme (WFP) in overcoming micronutrient deficiencies in nutritionally-vulnerable groups and low-income food-deficit countries continue to be thwarted by challenges such as technical and managerial capacity constraints, the need for systematic compliance with procurement specifications and quality control, clearer policies on micronutrient content labeling, and the need for cash resources to support many aspects associated with local processing and fortification activities [57]. 

### 3.5. Recommendations

The World Health Organization has recommended that weekly iron and folic acid supplementation should be considered as a strategy for prevention of iron deficiency in population groups. This is particularly so where the prevalence of anemia is above 20% among women of reproductive age [58]. Since iron tablets induce a high concentration of free radicals in the intestinal milieu, which may damage the intestinal epithelium, the minimal essential iron dose is to be recommended [59]. Therefore, there is a need to establish effective and safe doses of supplemental iron with folic acid either as daily or weekly supplementation; this should take into consideration nutritional and haematological status of women in developing countries. Though compliance with weekly dosage may be better than daily regimen because of reduced side effects, it is desirable to conduct further field randomized controlled trials in order to establish the efficacy of weekly supplementation compared to daily regimen.

Alternatively, an equally effective, safe, and affordable iron compound with little or no side effect can be developed for use especially in public health antenatal supplementation programmes. To this end, we recommend a cue to be taken from new approaches to iron fortification technology development whereby iron-mediated undesirable taste and appearance are prevented while its stability and bioavailability are preserved [60]. While this approach is being pursued, we recommend that appropriate measures be taken to strengthen the existing health systems in dealing with gastro-intestinal and life-threatening side effects of iron supplements.

Thus, clinical skills of local health staff could be improved through targeted trainings such as the WHO training on Life Saving Skills [61] for health workers. Though the training lays emphasis on core midwifery skills, it could be expanded to include cardiopulmonary resuscitation of mothers; furthermore, local health staff should be provided with appropriate state-of-art equipment to work with. Although, laboratories at the health facilities in developing countries usually lack the capacity to conduct quality blood test, they could be strengthened to provide reliable estimations of red blood cell indices such as haemoglobin, haematocrit, mean cell volume, mean cell haemoglobin, and mean cell haemoglobin concentration. Where possible, estimation of serum ferritin level is desirable to adjust the dose of iron supplements [62]. With respect to partial implementation of preventive treatment, local health staff should be encouraged on adherence to the guidelines on antenatal care package at any given antenatal visit so that partial implementation of preventive treatments is minimized.

Mass fortification programme of common local staple foods with iron and folic acid is a long-term goal, which national governments in developing countries should consider as a strategy aimed at reducing the prevalence of iron deficiency anaemia in the general population. Since iron deficiency anaemia in pregnancy is determined by preexisting body iron stores, among other factors, we recommend that the mass fortification programme should be located and implemented within the context of reproductive health services.

## 4. Conclusions

Iron deficiency remains the most important cause of anaemia in pregnancy in developing countries. Hence, its contribution to increased risks of low birth weight, prematurity, and maternal morbidity cannot be underscored. Prophylaxis iron supplement and food fortification with iron have the prospects of improving maternal and child health, except for the identified constraints. Sustained advocacy in tackling micronutrient deficiencies at national and international policy levels is also a prerequisite to the attainment of Millennium Development Goals 4 and 5.

## Figures and Tables

**Table 1:** A list of randomised and quasi-randomised trials used for review.

	Study	Study site	Subject number	Study question	Study methods	Study outcome
(1)	Zamani et al., 2008 [8]	Iran	152 pregnant women	Twice weekly iron supplementation versus daily regimen	Randomised control trial	Haemoglobin concentration
(2)	Bencaiova et al., 2009 [25]	Switzerland	260 pregnant women	intravenous iron sucrose versusdaily oral ferrous sulphate	Randomised control trial	Haemoglobin concentration; iron stores
(3)	Asibey-Berko et al., 2007 [29]	Ghana	184 women	Double-fortified salt versus weekly oral iron supplement versus weekly placebo	Double-blind randomisedcontrolled trial	Haemoglobin concentration
(4)	Hoa et al., 2005 [31]	Vietnam	168 women	Milk fortified with iron versus milk nonfortified with iron versus iron supplementation versus placebo	Quasirandomised trial	Haemoglobin concentration
(5)	Young et al., 2000 [36]	Malawi	413 pregnant women	Daily regimen versus weekly iron supplementation	Randomised controlled trial	Haemoglobin concentration
(6)	Latham et al., 2003 [30]	Tanzania	Pregnant women	Micronutrient dietary supplementversus placebo	Randomised controlled trial	Iron stores
